# Loss of the *Mecp2* gene in parvalbumin interneurons leads to an inhibitory deficit in the amygdala and affects its functional connectivity

**DOI:** 10.1186/s13229-025-00699-5

**Published:** 2026-01-05

**Authors:** Maj Liiwand, Joni Haikonen, Bojana Kokinovic, Svetlana M. Molchanova, Teemu Aitta-aho, Sari E. Lauri, Maria Ryazantseva

**Affiliations:** 1 Neuroscience Center, Helsinki Institute of Life Science, University of Helsinki, Helsinki, Finland; 2 Molecular and Integrative Biosciences Research Programme, University of Helsinki, Helsinki, Finland; 3 Department of Pharmacology, University of Helsinki, Helsinki, Finland

**Keywords:** MECP2, Rett syndrome, Amygdala, Parvalbumin interneurons

## Abstract

**Background:**

The *MECP2* gene is located on the X chromosome and encodes a methyl-CpG-binding protein 2 involved in transcriptional regulation. Loss-of-function mutations in the *MECP2* gene lead to Rett syndrome, a severe neurodevelopmental disorder. The clinical picture of Rett syndrome includes, among other symptoms, social deficits, learning impairment, and heightened anxiety. The amygdala is a brain region responsible for emotional learning and is involved in the regulation of social behaviour as well as fear and anxiety. Parvalbumin interneurons tightly control the excitability, oscillation and synchronisation of the amygdala network, which are relevant to its functions. Here, we investigated the effects of *Mecp2* gene ablation in parvalbumin interneurons on the microcircuit and functional connectivity of the mouse amygdala.

**Methods:**

Male mice with conditional knockout of the *Mecp2* gene in parvalbumin interneurons were used as a genetic mouse model. Littermates with an intact gene were used as controls. Ex vivo brain slice electrophysiology, combined with pharmacology and optogenetics, was utilised to characterise microcircuits within the lateral amygdala. In vivo functional ultrasound imaging was used to visualise the connectivity within the amygdala–ventral hippocampus–prefrontal cortex network triad.

**Results:**

Loss of *Mecp2* in parvalbumin interneurons significantly attenuated GABAergic synaptic input to principal neurons in the lateral amygdala. The deficit in inhibition was accompanied by higher excitability of local principal neurons in adult animals. A deficient in vivo functional connectivity of the amygdala with the ventral hippocampus and prefrontal cortex was observed in conditional knockouts.

**Limitations:**

This study used only male mice. *Mecp2* knockout males exhibit shorter latency to symptom onset and lower phenotypic variability, making them suitable for mechanistic studies. Since previous studies in the field used males, we aimed to advance the existing body of research using the same approach. Finally, the link between the effects observed and possible behavioural alterations needs further investigation.

**Conclusions:**

Our study characterised the consequences of *Mecp2* loss in parvalbumin interneurons on amygdala microcircuit function and connectivity within the prefrontal cortex‒amygdala‒hippocampus triad. It also provided evidence that supports and complements previous findings on the role of interneurons in the functional deficits observed in *Mecp2* knockout animal models.

**Supplementary information:**

The online version contains supplementary material available at 10.1186/s13229-025-00699-5.

## Background

The *Methyl CpG binding protein 2 (MECP2)* gene, located on the X chromosome, encodes a transcriptional regulator protein Mecp2 and is associated with Rett syndrome and *MECP2* duplication syndrome [1, 2]. These neurodevelopmental disorders significantly affect brain development and lead to the early emergence of symptoms, including anxiety, disrupted social interaction skills, and learning impairments [1–4]. Similar symptoms are reported in autism spectrum disorders (ASDs), in which mutations in the *MECP2* gene have also been described [3]. The biology behind these symptoms, however, is poorly understood.

A significant number of endophenotypes, including social and learning deficits, associated with *MECP2* dysfunction in patients can be replicated in mice by depleting the *Mecp2* gene in GABAergic neurons [5, 6]. Further studies have shown that conditional knockout of the *Mecp2* gene in parvalbumin (PV)-type GABAergic interneurons specifically leads to alterations in motor, sensory, social, and cognitive functions [7]. The conditional knockout of only one *Mecp2* allele in the PV interneurons of mice results in deficiencies in cortical plasticity and related behavioural features [8]. Although these results highlight the importance of *Mecp2* for the function of PV interneurons and connect the neurological consequences to compromised GABAergic signalling, they do not provide a full picture of the circuit abnormalities in affected brain areas.

PV interneurons are fast-spiking GABAergic neurons that provide critical inhibitory control and support activity synchronisation in the cortex and cortical-like structures such as the hippocampus and basolateral part of the amygdala. These neurons are highly vulnerable to stressors and have been implicated in many psychiatric diseases and in neurodevelopmental syndromes with an ASD component [9, 10]. Knockout of the PV gene or a reduction in PV interneuron numbers leads to a robust ASD-like behavioural phenotype in animal models, including impaired social interaction and deficits in learning [9–11].

Mice lacking the *Mecp2* gene in PV interneurons demonstrated learning and memory deficits in fear conditioning [7]. Excitatory projection neurons in the basolateral nucleus of the amygdala (BLA) are subject to fear learning-associated plasticity [12]. Their excitability and activity in fear acquisition and expression are regulated by PV interneurons through cue-related inhibition and disinhibition. PV interneurons in the lateral (LA) part, but not in the basal part (BA), of the BLA possess complex dendritic arborisation, receive potent excitatory drive, and mediate feedforward inhibition of principal neurons (PNs) [13]. In a fear conditioning trial during the presentation of an unconditional stimulus, a bidirectional role of PV interneurons has been shown: silencing augments fear learning, whereas activation reduces fear learning [13–15]. After fear conditioning, PV neurons in the LA exhibit a persistent reduction in their excitatory input and inhibitory output, which is indicative of the plasticity not observed in the BA [13]. The effect of *Mecp2* loss in PV interneurons on the LA microcircuit has never been studied. However, knockout of *Mecp2* in other brain areas has previously been demonstrated to affect the excitatory input to [16, 17] or the excitability of [18] PV interneurons.

We hypothesised that the loss of *Mecp2* influences the synaptic connectivity or excitability of PV interneurons in the LA, leading to alterations in the excitability of PNs and perturbing the functional connectivity of the amygdala. Here, we compared the synaptic and excitatory properties of mouse LA local PV interneurons lacking the *Mecp2* gene to those with the intact gene. We further investigated the properties of excitatory PNs in the LA and tested for deficits in fast and slow GABAergic inhibition. Moreover, we tested the functional connectivity of the amygdala in vivo to detect the consequences of genetic ablation of the *Mecp2* gene in PV interneurons for BLA-ventral hippocampus (vHPC) and BLA-medial prefrontal cortex (mPFC) circuits. The results of our research demonstrated that the *Mecp2* gene is required for PV interneurons to effectively control local inhibition and sustain functional connectivity in the amygdala.

## Methods

### Animals

*The* B6.129P2-^*Mecp2tm1bird*^/J (Mecp2^fl/fl^ [19]) mouse line was crossed with the B6.129P2-*Pvalb*^tm1(cre)Arbr/J^ (PV-Cre, JAX 008069) line to produce ablation of the *Mecp2* gene selectively in parvalbumin interneurons (PV-Cre::Mecp2^fl/y^). The littermates of the PV-Cre::Mecp2^x/y^ genotype, referred to as PV-Cre, were used as controls. The mice were housed in individually ventilated cages with a 12-h light/12-h dark cycle (lights off at 7:00 p.m. − 7:00 a.m.), and food and water were supplied *ad libitum*. All animal experiments were performed following the University of Helsinki Animal Welfare Guidelines and approved by the National Animal Experiment Board of Finland (license numbers: KEK21-029, ESAVI/20432/2022). 1.5–2-months-old male mice were used for experiments with adult animals, with the control and knockout groups having equal age distributions. Male pups were used at postnatal day 12–14 (P12-14) and 21–25 (P21-25).

### Immunohistochemistry (IHC)

Mice were transcardially perfused with PBS and 4% PFA under deep anaesthesia. The brains were extracted and placed in 4% PFA overnight at 4 °C, after which they were washed twice with PBS. 50-µm-thick coronal sections were cut using a vibratome (Vibratome 1000 Plus) and placed in 12-well plates. The sections were incubated in a blocking solution (10% goat serum and 0.3% Triton-X100 in PBS) at room temperature (RT) for 1 hour, then washed with a wash buffer (2% goat serum and 0.06% Triton-X100 in PBS) for 20 min at RT, and incubated with primary antibodies diluted into the wash buffer (1:1000 PV235 mouse (SWANT), 1:1000 Mecp2 chicken (Novus)) overnight at 4 °C. The next day, the sections were washed with the wash buffer 2 × 20 min at RT, incubated in secondary antibodies diluted into the wash buffer (1:1000 Alexa 488 goat anti-chicken (Life Technologies), 1:1000 Alexa 594 goat anti-mouse (Life Technologies)) for 2 hours at RT, finally washed with PBS 3 × 20 min at RT, and mounted onto microscope slides using Vectashield mounting medium. The stained sections were imaged with Zeiss Axio Imager.M2 microscope equipped with ApoTome 2 structured illumination slider, 20X Plan objective and CMOS camera (Hamamatsu ORCA Flash 4.0 V2) and Zen Blue software. Images were analysed with Imaris (RRID:SCR_007370) and Fiji software [20].

### Viral injections

AAV viral vectors encoding floxed EGFP (AAV8; pAAV-hSyn-DIO-EGFP; 2.2 × 10^13^ GC/ml, Addgene #50457) or floxed channelrhodopsin-2 (ChR2, AAV1; pAAV-EF1a-double floxed-hChR2(H134R)-mCherry-WPRE-HGHpA; 1 × 10^13^ GC/ml, Addgene #20297) were bilaterally injected into the BLA of adult mice, as described before [21]. In short, mice were anesthetized with isoflurane (4% induction, 1.5–2% maintenance in O_2_/CO_2_) and placed in a stereotaxic frame (StoelTing) with body temperature maintained at 37 °C. A glass micropipette (Clunbury Scientific LLC, tip ~20–30 μm) connected to a 10 μL Hamilton syringe (WPI) mounted on a microinjection pump (UMP3-4, WPI) was lowered into the basolateral amygdala using coordinates from bregma and dura: (1) AP −1,8, ML 3.4–3.5, DV 4.1–4.3; (2) AP −2.3, ML 3.4–3.5, DV 4.1–4.3. Undiluted AAV vector was injected bilaterally (4 injections per hemisphere, 0.1 μL per site). Experiments were performed > 2 weeks after injection.

### Electrophysiology

Acute coronal sections were prepared as described previously [22]. Briefly, the brain was removed and immediately placed in carbonated (95% O_2_/5% CO_2_) ice-cold N-methyl-D-glucamine (NMDG)-based protective cutting solution (pH 7.3–7.4) containing (in mM) 92 NMDG, 2.5 KCl, 1.25 NaH_2_PO_4_, 30 NaHCO_3_, 20 HEPES, 25 glucose, 2 thiourea, 5 Na-ascorbate, 3 Na-pyruvate, 0.5 CaCl_2_ and 10 MgSO_4_ [23]. A vibratome (Leica VT 1200S) was used to obtain 300-µm-thick brain slices. Slices containing the amygdala were placed into a slice holder and incubated for 8–10 min at 34 °C in the NMDG–based solution. Slices were then transferred into a separate slice holder at room temperature with a solution containing (in mM): 92 NaCl, 2.5 KCl, 1.25 NaH_2_PO_4_, 30 NaHCO_3_, 20 HEPES, 25 glucose, 2 thiourea, 5 Na-ascorbate, 3 Na-pyruvate, 2 CaCl_2_ and 2 MgSO_4_ (saturated with 95% O_2_/5% CO_2_). After 1–4 h of recovery, the slices were placed in a submerged heated (30–32°C) recording chamber and continuously perfused with standard ACSF containing (in mM): 124 NaCl, 3 KCl, 1.25 NaH_2_PO_4_, 26 NaHCO_3_, 15 glucose, 1 MgSO_4_ and 2 CaCl_2_.

*Whole-cell patch-clamp recordings* were done from amygdala neurons under visual guidance using glass capillary microelectrodes with a resistance of 3.5–5.5 MΩ. A Multiclamp 700B amplifier (Molecular Devices), Digidata 1322 (Molecular Devices) or NI USB-6341 A/D board (National Instruments), and WinLTP version 2.20 or pClamp 11.0 software were used for data collection, with a low-pass filter (10 kHz) and a sampling rate of 20 kHz. Whole-cell current-clamp recordings of membrane excitability in PNs and PV interneurons in the LA were performed with the following filling solution (in mM): 135 K-gluconate, 10 HEPES, 5 EGTA, 2 KCl, 2 Ca(OH)_2_, 4 Mg-ATP, and 0.5 Na_2_-GTP (280 mOsm, pH 7.2). The resting membrane potential was sampled and then adjusted to −70 mV. Depolarising current steps of 500-ms (PV interneurons) or 1000-ms duration (PNs) were applied to induce action potential firing. The amplitude of the injected current was increased in 5–10 pA increments. Drug-induced GABA_B_-mediated and G protein-coupled inwardly rectifying potassium channel (GIRK) currents were recorded from LA PNs with the same solutions in the presence of antagonists for NMDA and AMPA receptors (50 µM AP-5 and 50 µM NBQX, respectively). Picrotoxin (100 µM) was included in the ACSF to block GABA_A_ receptors, and the holding potential of the neurons was −50 mV. A valve control system (VC-6-PINCH, Warner Instruments) with a manifold adjusted to the perfusion tube was used for fast local drug application. ACSF, including the abovementioned antagonists, was applied directly to the LA to record a baseline before switching to a solution supplemented with the relevant drug (25 µM SKF97541 or 2 mM barium chloride). Stimulation-induced GABA_B_ postsynaptic response (slow IPSC) recordings were performed as previously described [24, 25], with a bipolar stimulating electrode placed at the apical cortical edge of the lateral amygdala (LA), whereas the PN for the recording was approximately at the same depth in the middle of the lateral amygdala. Stimulation responses were evoked by a stimulus isolation unit (DS2A, Digitimer Ltd.) by applying a short burst of four stimuli at 100 Hz in the presence of picrotoxin, NBQX, and AP-5 at a holding potential of − 50 mV. The stimulation power was 28–32 V and was adjusted in this range to reach maximum response amplitudes. After the stimulation was not able to induce a larger current, the V value was used for stimulation and recording of a series of responses. The GABA_B_ antagonist 5 μM CGP 55845 was added to the recording chamber through the main ASCF flow. Spontaneous synaptic currents (sEPSCs and sIPSCs) in PV interneurons and LA PNs were recorded under a whole-cell voltage-clamp mode with a filling solution containing (in mM) 135 K-gluconate, 10 HEPES, 5 EGTA, 2 KCl, 2 Ca(OH)_2_, 4 Mg-ATP, and 0.5 Na_2_-GTP at a holding potential of −50 mV. For light-induced IPSCs, recordings were performed using a high-chloride intracellular solution containing (in mM): 130 CsCl, 10 HEPES, 0.5 EGTA, 8 NaCl, 4 Mg-ATP, 0.3 Na_2_-GTP, and 5 QX314 (280 mOsm, pH 7.2) at a −70 mV holding potential in the presence of NBQX and AP-5. Optical stimulation of PV interneurons was performed using a pE-300 LED system (CoolLED). Short pulses (0.5 ms, 50 ms interval) of 470 nm blue light were used for paired-pulse stimulation. WinLTP software was used to calculate the peak amplitude of the evoked synaptic responses. For analysis of the paired-pulse ratio (PPR), 7–10 responses were averaged in each experimental condition. The PPR was calculated as the amplitude ratio of response 2/response 1. The frequency and amplitude of spontaneous synaptic events were analysed using the MiniAnalysis 6.0.3 program. sIPSCs and sEPSCs were identified in the analysis as outward or inward currents (for IPSCs depending on experimental conditions) with typical kinetics, respectively, that were at least 3 times the amplitude of the baseline level of noise. For the pooled data, averages for the baseline data were calculated over a 10-minute period. Action potential frequencies were analysed via the threshold search algorithm in Clampfit software. The AP half-width potential was analysed from the 3^rd^ spike in the train.

### Confocal microscopy and spine density analysis

Biocytin (0.2%, HelloBio) was added to the filling solution. For post-hoc morphological characterisation of the biocytin-filled neurons, the slices were fixed overnight in 4% paraformaldehyde (4 °C), after which they were washed with cold phosphate-buffered saline (PBS) and permeabilised with 0.3% Triton-X 100 (Sigma‒Aldrich) in PBS overnight at 4 °C. Alexa Fluor 568 streptavidin (1:500; Life Technologies) was added to the permeabilisation solution, which was subsequently incubated overnight at 4 °C. The PBS-washed slices were mounted onto slides and blind-coded for morphological analysis. Dendritic spines were imaged via an LSM Zeiss 710 confocal microscope (Zeiss alpha Plan-Apochromat 63x/1.46 OilKorr M27 objective). The spines were imaged with a resolution x, y = 0.066 µm and Z-stack interval of 0.5 μm. Spine density was evaluated on the 3 to 4 secondary dendrites per cell; both apical and basal dendrites were included into the analysis, as there is no difference in spine density between proximal apical dendrites and proximal basal dendrites of BLA principal cells [26]. Actual spine detection was done using NeuronStudio software to quantify spines in a 2D maximal intensity-projection Z-stack image. Verification of spine detection was performed semi-manually. Only clearly visible spines that extend laterally from a dendrite were counted [27]. The averaged values of spine density per cell were used for statistical analysis.

### In vivo functional ultrasound (fUS)

Resting-state functional connectivity between the medial prefrontal cortex (mPFC), basolateral amygdala (BLA) and ventral hippocampus (vHPC) in littermates from 3 litters was assessed using the Iconeus One functional ultrasound imaging system. The animals were anaesthetised with medetomidine (1 mg/kg) + ketamine (75 mg/kg), after which the head fur was shaved. The animal head was fixed in a stereotaxic frame with a heating pad (35 °C), and ultrasound gel was spread on the scalp. The probe was controlled using the IcoScan (v. 1.3.1) software, first placed near the scalp and then adjusted to visualise the posterior cerebral arteries (PCA). A coronal mapping (Angio3D) scan was performed from the PCA to the mPFC with a slice interval of 0.1 mm. The scan was uploaded to IcoStudio (v. 1.2.1) software and mapped via the automated mapping system. Regions of interest were selected (the mPFC, BLA, and vHPC), and markers were placed on the map. Probe coordinates were computed based on these markers to align all areas of interest in a single sagittal plane. The coordinates were manually uploaded to the IcoScan software, and a 20-minute 2D fUS scan was performed. After the scan, the animals were placed into a heat chamber (35 °C) and injected with atipamezole (0.5 mg/kg) for recovery. IcoStudio software was used to compute a functional connectivity matrix based on the fUS scan. Baseline correction and a 0.2 Hz low-pass filter were applied to the final results. For statistical analysis, the matrix values (Pearson’s correlation coefficients) were transformed using Fisher’s z-transformation. The size of effects on connectivity between brain areas’ nodes was analysed as a Cohen’d with R software (Package ‘effsize’) [28]. The chord diagram for effect sizes on connectivity was done with the “chordDiagram()” function and “circlize” package in R [29].

## Results

### PV neurons lacking the *Mecp2* gene receive higher amplitudes of excitatory synaptic currents but release less GABA to principal neurons in the lateral amygdala

We used a previously published strategy for Cre-dependent conditional knockout of the floxed *Mecp2* gene in the PV interneurons of the mouse brain [7, 18]. PV-Cre males were crossed with Mecp2^fl/wt^ females to obtain littermates with conditional knockout of *Mecp2* in PV interneurons (PV-Cre::Mecp2^fl/y^) and with an intact *Mecp2* gene (PV-Cre::Mecp2^wt/y^, which will be referred to as PV-Cre according to original studies [7, 18]). The majority of PV neurons (PV+; neurons stained with PV antibody) in mPFC, vHPC, and amygdala of adult PV-Cre::Mecp2^fl/y^ mice lack MeCP2 protein expression (Supplementary Fig. 1a). However, as demonstrated previously [18], around 30% of PV interneurons were positive for MeCP2 protein in the regions we had evaluated after conditional knockout (Supplementary Fig. 1a and b).

**Fig. 1:**
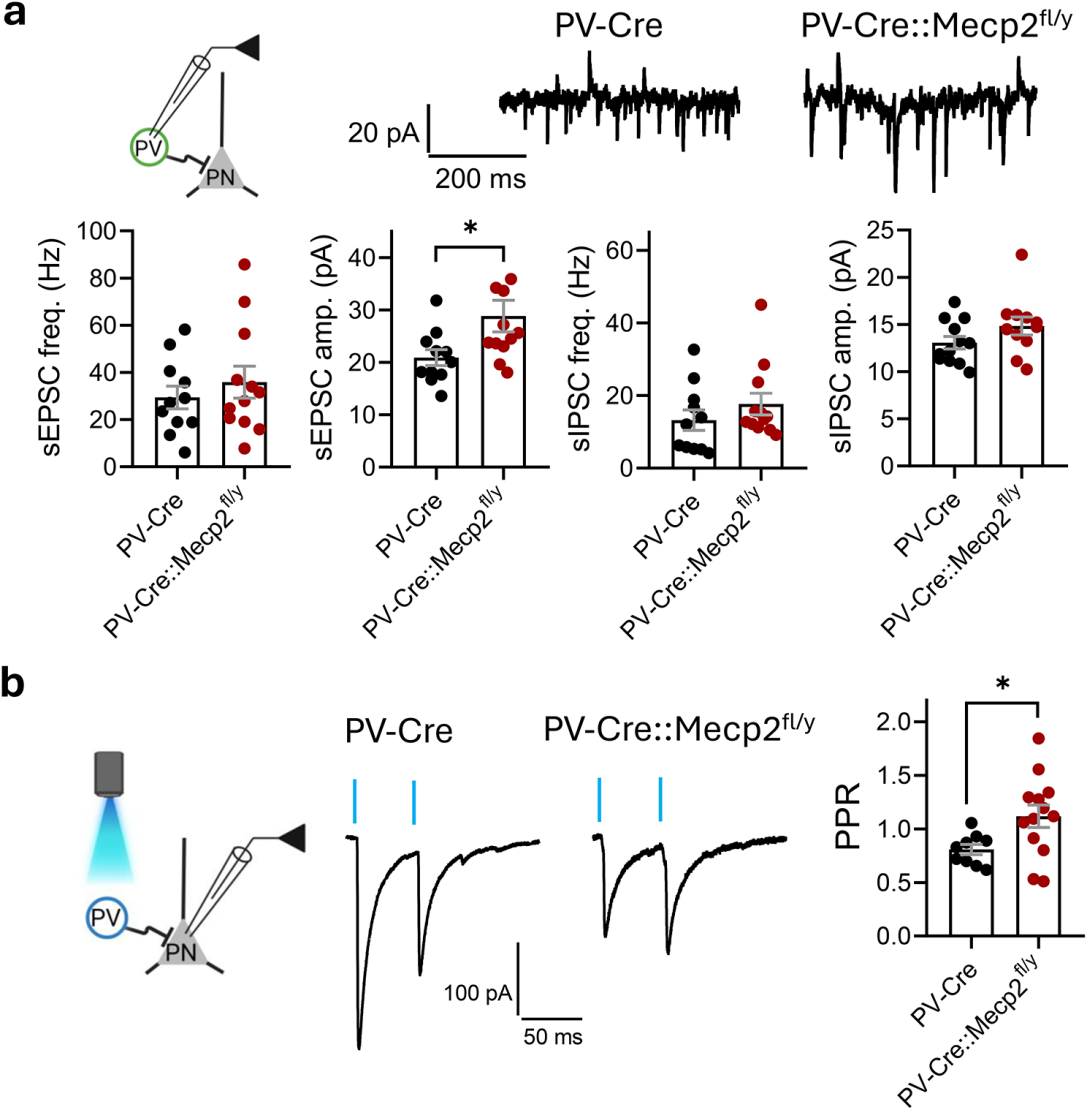
*Mecp2* ablation in PV interneurons leads to post- and presynaptic abnormalities in the lateral amygdala. **a**. Experimental scheme: PV – EGFP-expressing PV interneuron, PN – principal neuron. Example traces of whole-cell recordings of synaptic currents at a holding potential of −50 mV performed in the PV interneurons within the lateral amygdala of PV-Cre and PV-Cre::Mecp2^fl/y^ littermates (*n* = 11 neurons from 3 mice for PV-Cre; *n* = 12 neurons from 3 mice for PV-Cre::Mecp2^fl/y^). The frequencies and amplitudes of sEPSCs (freq.: t test, *t* = 0.7666, df = 21, *p* = 0.4519; ampl.: t test, *t* = 2.286, df = 21, *p = 0.0327). Frequencies and amplitudes of sIPSCs (freq.: t test, *t* = 1.075, df = 21, *p* = 0.2948; ampl.: t test, *t* = 1.542, df = 21, *p* = 0.1381). **b.** Experimental scheme: PV – ChR2-expressing PV interneuron, PN – principal neuron. Paired-pulse ratios of optogenetically induced (50 ms interval) inhibitory synaptic responses recorded from PNs within the lateral amygdala of PV-Cre and PV-Cre::Mecp2^fl/y^ littermates expressing ChR2 in the PV interneurons (*n* = 9 neurons from 4 mice for PV-Cre; *n* = 13 neurons from 3 mice for PV-Cre::Mecp2^fl/y^; t test, *t* = 2.349, df = 20, *p = 0.0292). All the data are shown as the mean ± S.E.M

To visualise the PV interneurons for the electrophysiological characterisation in the LA, the mice were locally injected with an adeno-associated virus (AAV) construct encoding Cre-dependent EGFP. The synaptic properties and excitability of PV interneurons were then addressed in acute coronal slices containing the BLA via whole-cell patch-clamp recording. sEPSCs from EGFP-labelled neurons in the LA were recorded as inward currents when the recording was performed at a −50 mV holding potential with a low-chloride pipette solution (Fig. 1a). The frequencies of sEPSCs were not different for PV interneurons among genotypes, whereas the amplitudes of sEPSCs were significantly higher in PV interneurons lacking *Mecp2* (freq.: 29.41 ± 4.823 Hz for PV-Cre vs. 35.87 ± 6.735 Hz for PV-Cre::Mecp2^fl/y^, t test, *t* = 0.7666, df = 21, *p* = 0.4519; ampl.: 20.90 ± 1.510 pA for PV-Cre vs. 28.83 ± 3.015 pA for PV-Cre::Mecp2^fl/y^, t test, *t* = 2.286, df = 21, *p* = 0.0327) (Fig. 1b). sIPSCs were recorded simultaneously as outward currents at a holding potential of −50 mV. No differences were observed in the frequencies or amplitudes of sIPSCs in PV interneurons among the genotypes (freq.: 13.25 ± 2.810 Hz for PV-Cre vs. 17.66 ± 2.966 Hz for PV-Cre::Mecp2^fl/y^, t test, *t* = 1.075, df = 21, *p* = 0.2948; ampl.: 13.07 ± 0.6627 for PV-Cre vs. 14.83 ± 0.9525 for PV-Cre::Mecp2^fl/y^, t test, *t* = 1.542, df = 21, *p* = 0.1381). Therefore, the only difference in the spontaneous synaptic input of PV interneurons lacking the *Mecp2* gene was the sEPSCs amplitude values.

In addition to the input signal, we evaluated the synaptic output of PV interneurons lacking *Mecp2*. For that purpose, we addressed GABA release from PV interneurons to PNs in the LA. The AAV viral vector encoding Cre-dependent ChR2 was locally injected into the BLA, and blue light was used to induce the synaptic release of GABA from PV interneurons in brain slices. Opto-IPSCs were recorded in whole-cell mode from PNs in the LA with a high-chloride pipette solution and a −80 mV holding potential. NBQX and AP-5 were used to block AMPA and NMDA currents, respectively. A paired-pulse protocol was applied to address the probability of GABA release. The paired-pulse ratio of IPSCs in PNs in the LA of PV-Cre littermates was significantly lower than that in PNs in the LA of PV-Cre::Mecp2^fl/y^ mice (PPR: 0.8074 ± 0.04814 for PV-Cre vs. 1.117 ± 0.1040 for PV-Cre::Mecp2^fl/y^, t test, *t* = 2.349, df = 20, *p* = 0.0292) (Fig. 1b), indicating a lower probability of GABA release from PV interneurons lacking *Mecp2*.

### Loss of the *Mecp2* gene in PV interneurons within the LA does not affect their excitability

**Fig. 2:**
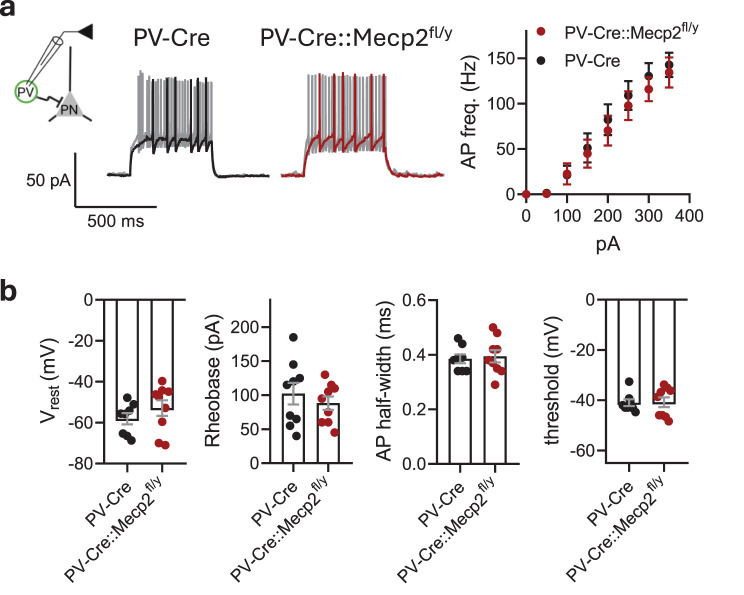
*Mecp2* gene loss in parvalbumin interneurons of the lateral amygdala does not affect their excitability. **a.** Experimental scheme: PV – EGFP-expressing PV interneuron, PN – principal neuron. Action potential (AP) firing frequencies in response to current steps plotted from the rheobase with a 25 pA increment (*n* = 8 neurons from 3 mice for PV-Cre; *n* = 9 neurons from 3 mice for PV-Cre::Mecp2^fl/y^; two-way RM ANOVA, genotype effect: F(1, 15) = 0.1802, *p* = 0.6773) and example traces of AP bursts in response to 25 and 100 pA current steps. **b.** Resting membrane potential (V_rest_), rheobase, AP half-width and firing threshold for PV interneurons in the lateral amygdala of PV-Cre and PV-Cre::Mecp2^fl/y^ littermates (*n* = 8 neurons from 3 mice for PV-Cre; *n* = 9 neurons from 3 mice for PV-Cre::Mecp2^fl/y^; V_rest_: t test, *t* = 1.086, df = 15, *p* = 0.2945; rheobase: t test, *t* = 0.7438, df = 16, *p* = 0.4678; AP half-width: t test, *t* = 0.3326, df = 15, *p* = 0.7440; threshold: t test, *t* = 0.08054, df = 15, *p* = 0.9369). All the data are shown as the mean ± S.E.M

The passive membrane properties and intrinsic excitability of the EGFP-labelled PV interneurons in the LA were examined via the intracellular injection of step currents in current-clamp mode. There was no difference between the genotypes for action potential firing frequencies (two-way RM ANOVA, genotype effect: F (1,15) = 0.1802, *p* = 0.6773) (Fig. 2a), resting membrane potential (−58.11 ± 2.732 mV for PV-Cre vs. −52.89 ± 3.821 mV for PV-Cre::Mecp2^fl/y^, t test, *t* = 1.086, df = 15, *p* = 0.2945), rheobase (102.2 ± 15.90 pA for PV-Cre vs. 88.33 ± 9.789 pA for PV-Cre::Mecp2^fl/y^, t test, *t* = 0.7438, df = 16, *p* = 0.4678), action potential half-width (0.3850 ± 0.01626 ms for PV-Cre vs. 0.3944 ± 0.02249 ms for PV-Cre::Mecp2^fl/y^, t test, *t* = 0.3326, df = 15, *p* = 0.7440), or firing threshold (−41.00 ± 1.345 mV for PV-Cre vs. −40.80 ± 1.929 mV for PV-Cre::Mecp2^fl/y^, t test, *t* = 0.08054, df = 15, *p* = 0.9369) (Fig. 2b).

### Principal neurons in the LA of mice lacking *Mecp2* in PV interneurons demonstrate increased excitability

**Fig. 3:**
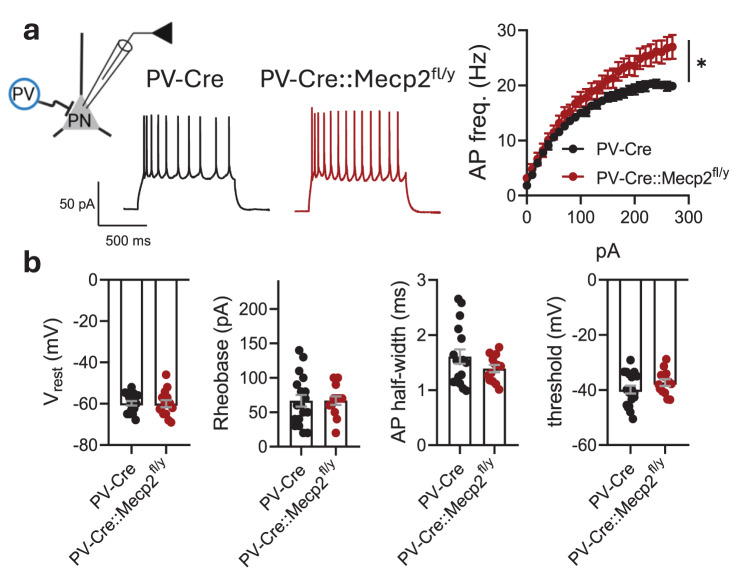
*Mecp2* gene loss in PV interneurons increases the excitability of principal neurons in the lateral amygdala. **a.** Scheme of the experiment: PV – PV interneuron, PN – principal neuron. Action potential (AP) firing frequencies of PNs in response to current steps plotted from the rheobase with a 10 pA increment (*n* = 18 neurons from 5 mice for PV-Cre; *n* = 13 neurons from 3 mice for PV-Cre::Mecp2^fl/y^; two-way RM ANOVA, genotype effect: F (1, 29) = 5.217, *p = 0.0299) and example traces of AP bursts in response to a 150 pA current step. **b.** Resting membrane potential (V_rest_), rheobase, AP half-width and firing threshold for PNs in the lateral amygdala of PV-Cre and PV-Cre::Mecp2^fl/y^ littermates (*n* = 18 neurons from 5 mice for PV-Cre; *n* = 13 neurons from 3 mice for PV-Cre::Mecp2^fl/y^; V_rest_: t test, *t* = 0.09421, df = 30, *p* = 0.9256; rheobase: t test, *t* = 0.02248, df = 29, *p* = 0.9822; ap half-width: t test, *t* = 1.373, df = 28, *p* = 0.1806; threshold: t test, *t* = 1.300, df = 29, *p* = 0.2038). All the data are shown as the mean ± S.E.M

PV interneurons potently modulate PNs excitability in the amygdala through perisomatic innervation [30]. PNs in the LA of PV-Cre::Mecp2^fl/y^ had higher action potential firing frequencies recorded with the application of step currents in the current-clamp mode (two-way RM ANOVA, genotype effect: F (1, 29) = 5.217, *p = 0.0299) (Fig. 3a). At the same time, there were no differences in the resting membrane potential (−59.82 ± 1.096 mV for PV-Cre vs. −60.00 ± 1.701 mV for PV-Cre::Mecp2^fl/y^, t test, *t* = 0.09421, df = 30, *p* = 0.9256), firing threshold (−39.87 ± 1.432 mV for PV-Cre vs. −37.29 ± 1.227 mV for PV-Cre::Mecp2^fl/y^, t test, *t* = 1.300, df = 29, *p* = 0.2038), action potential half-width (1.609 ± 0.1302 ms for PV-Cre vs. 1.389 ± 0.06622 ms PV-Cre::Mecp2^fl/y^, t test, *t* = 0.373, df = 28, *p* = 0.1806) or rheobase (66.67 ± 8.556 pA for PV-Cre vs. 66.92 ± 6.241 pA for PV-Cre::Mecp2^fl/y^, t test, *t* = 0.02248, df = 29, *p* = 0.9822) (Fig. 3b).

### Loss of *Mecp2* in PV interneurons leads to an inhibitory deficit in the LA, affecting both fast and slow inhibition

**Fig. 4:**
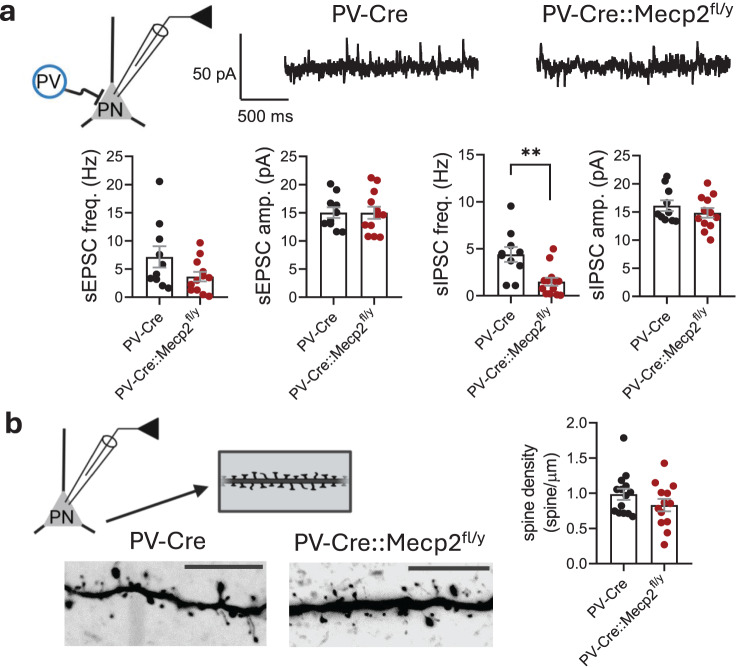
*Mecp2* gene loss in parvalbumin interneurons reduces fast inhibitory input to principal neurons in the lateral amygdala. **a.** Scheme of the experiment: PV – PV interneuron and PN – principal neuron. Example traces of whole-cell recordings of synaptic currents in the PNs within the lateral amygdala of PV-Cre and PV-Cre::Mecp2^fl/y^ littermates (*n* = 10 neurons from 4 mice for PV-Cre; *n* = 12 neurons from 3 mice for PV-Cre::Mecp2^fl/y^). Frequencies and amplitudes of sEPSCs (freq.: t test, *t* = 1.783, df = 20, *p* = 0.0897; ampl.: t test, *t* = 0.01377, df = 20, *p* = 0.9891). The frequencies and amplitudes of sIPSCs (freq.: t test, *t* = 3.470, df = 21, **p = 0.0023; ampl.: t test, *t* = 1.009, df = 20, *p* = 0.3250). **b.** Experimental scheme showing post hoc visualization of the dendritic spines of the patched neurons. Spine densities of PNs in the lateral amygdala (*n* = 14 neurons from 4 mice for the PV-Cre group; *n* = 13 neurons from 3 mice for the PV-Cre::Mecp2^fl/y^ group; Mann‒Whitney test, *U* = 70, *p* = 0.3255). Example images of spines of both genotypes; the scale bar represents 10 μm. All the data are shown as the mean ± S.E.M

To investigate whether *Mecp2* ablation from PV interneurons affects the overall synaptic input to LA PNs, sIPSCs and sEPSCs were recorded simultaneously in whole-cell voltage-clamp mode with a low-chloride pipette solution at a holding potential of −50 mV. There were no differences in the amplitudes or frequencies of sEPSCs between genotypes (freq.: 7.156 ± 1.893 Hz for PV-Cre vs. 3.662 ± 0.8540 Hz for PV-Cre::Mecp2^fl/y^, t test, *t* = 1.783, df = 20, *p* = 0.0897; ampl.: 15.02 ± 0.9445 pA for PV-Cre vs. 15.00 ± 1.082 for PV-Cre::Mecp2^fl/y^, t test, *t* = 0.01377, df = 20, *p* = 0.9891) (Fig. 4a). At the same time, a significantly lower frequency of sIPSCs was detected for PNs in the LA of PV-Cre::Mecp2^fl/y^ mice than in controls (freq.: 4.399 ± 0.7893 Hz for PV-Cre vs. 1.492 ± 0.4179 Hz for PV-Cre::Mecp2^fl/y^, t test, *t* = 3.470, df = 21, *p* = 0.0023) (Fig. 4a), which aligns with the lower probability of GABA release from PV interneurons. The amplitudes of sIPSCs were not different between the genotypes (amp.: 16.12 ± 0.9511 pA for PV-Cre vs. 14.83 ± 0.8562 pA for PV-Cre::Mecp2^fl/y^, *t* = 1.009, df = 20, *p* = 0.3250) (Fig. 4a). The lack of differences in sEPSCs among genotypes was additionally supported by the absence of visible alterations in the densities of the dendritic spines at PNs filled with biocytin during recording and analysed post hoc (0.9852 ± 0.08029 spine/μm for PV-Cre vs. 0.8314 ± 0.08733 spine/μm for PV-Cre::Mecp2^fl/y^, Mann‒Whitney test, *U* = 70, *p* = 0.3255) (Fig. 4b). This finding indicates that the loss of *Mecp2* in PV interneurons does not affect the excitatory postsynaptic connectivity of PNs in the LA but reduces their inhibitory input.

**Fig. 5:**
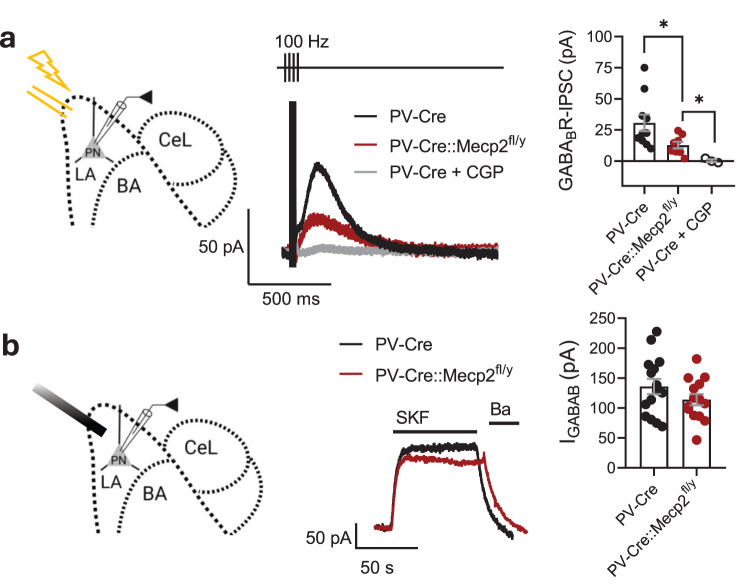
*Mecp2* gene loss in parvalbumin interneurons reduces slow inhibitory input to principal neurons in the lateral amygdala. **a.** Experimental scheme: stimulation of the LA nucleus of the amygdala with a bilateral electrode and recording from the PN in the LA. Amplitudes of stimulation-induced slow inhibitory postsynaptic currents in PNs of the lateral amygdala. CGP 55845 (5 μM, CGP) was applied to block GABA_B_R responses in PV-Cre mouse brain slices. (*n* = 10 neurons from 3 mice for PV-Cre; *n* = 8 neurons from 3 animals for PV-Cre:Mecp2^fl/y^; *n* = 3 neurons from 3 mice for CGP application; PV-Cre vs. PV-Cre::Mecp2^fl/y^: t test, *t* = 2.277, df = 16, *p = 0.0368; PV-Cre::Mecp2^fl/y^ vs. PV-Cre + CGP: t test, *t* = 2.662, df = 9, *p = 0.0260). Example traces of slow IPSCs in response to high-frequency stimulation (4 pulses, 10 ms interevent interval). **b.** Scheme of the experiment: fast solution application tube directed to the LA, recording from the PN in the LA. Amplitudes of outward currents induced by the application of the GABA_B_R agonist SKF 97541 (25 μM). (*n* = 15 neurons from 3 mice for PV-Cre; *n* = 14 neurons from 3 animals for PV-Cre::Mecp2^fl/y^; t test, *t* = 1.372, df = 27, *p* = 0.1814). Example traces of currents induced by the SKF and sensitive to 2 mM barium chloride (Ba). All the data are shown as the mean ± S.E.M

In addition to fast synaptic inhibition through GABA_A_ receptors, PNs in the LA receive a slow inhibition by the postsynaptic metabotropic GABA_B_ receptors, which activate GIRKs [24]. To assess slow inhibition, the release of GABA from local interneurons in the amygdala was activated with the stimulating electrode placed at the upper corner of the cortical side of the LA in brain slices (Fig. 5a). The response to local stimulation was not dependent on LA innervation by cortical inputs, as glutamatergic transmission was blocked with AMPA and NMDA receptor antagonists. The recording was performed from PNs in the middle of the LA in the presence of NBXQ, AP-5, and picrotoxin to block AMPA, NMDA, and GABA_A_ currents, respectively. The recording pipette contained a high-potassium solution. Repetitive high-frequency electrical stimuli (4 stimuli at 100 Hz) elicited a robust current at a −50 mV holding potential that was sensitive to a potent inhibitor of the GABA_B_ receptor CGP 55845 (Fig. 5a). The stimulation intensity was adjusted to induce the maximum amplitude of response when a further increase in intensity did not produce an additional rise in the current amplitudes. The maximum amplitude of the evoked GABA_B_ current was lower in the PNs of PV-Cre::Mecp2^fl/y^ mice than in those of controls (30.69 ± 6.649 pA for PV-Cre vs. 12.77 ± 2.743 pA for PV-Cre::Mecp2^fl/y^, t test, *t* = 2.277, df = 16, *p* = 0.0368) (Fig. 5a). However, fast application of the potent GABA_B_ receptor agonist SKF 97541 [31] induced comparable amplitudes of agonist-induced currents in PNs of both genotypes (135.9 ± 12.76 pA for PV-Cre vs. 113.9 ± 9.384 pA for PV-Cre::Mecp2^fl/y^, t test, *t* = 1.372, df = 27, *p* = 0.1814) (Fig. 5b). The lower amplitudes of electrically induced GABA_B_ currents align with the deficit of the inhibition provided by interneurons to PNs in the LA. As a potent GABA_B_ receptor agonist can still induce a current with an amplitude comparable to that of controls, there should be enough receptors and GIRK channels to provide slow inhibition when the GABA signal is sufficient.

### Inhibitory deficit in LA emerges in late adolescence in mice with *Mecp2* loss in PV interneurons

**Fig. 6:**
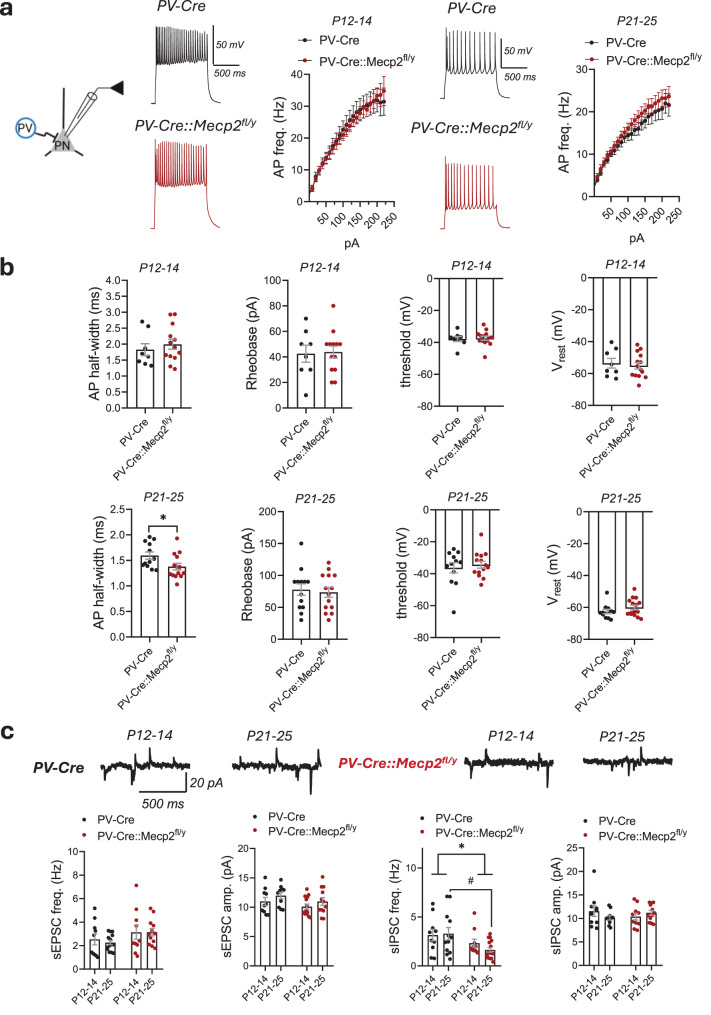
LA Inhibitory deficit driven by *Mecp2* loss in parvalbumin interneurons emerges during the third postnatal week. **a.** Scheme of the experiment: PV – PV interneuron, PN – principal neuron. Action potential (AP) firing frequencies of PNs at two time points (P12-14 and P21-25) in response to current steps plotted from the rheobase with a 10 pA increment (P12-14: *n* = 8 neurons/3 PV-Cre mice; *n* = 11 neurons/4 PV-Cre::Mecp2^fl/y^ mice; REML, genotype effect: F(1, 17) = 0.02818, *p* = 0.8687; P21-25: *n* = 15 neurons from 4 mice for PV-Cre; *n* = 16 neurons from 4 mice for PV-Cre::Mecp2^fl/y^; REML, genotype effect: F(1, 27) = 0.9815, *p* = 0.3306), example traces of AP bursts in response to a 200 pA current step **b.** AP half-width, rheobase, firing threshold and resting membrane potential (V_rest_) for PNs in the LA of PV-Cre and PV-Cre::Mecp2^fl/y^ mice at P12-14 and at P21-25 (P12-14: *n* = 8 neurons/3 PV-Cre mice; *n* = 13 neurons/4 PV-Cre::Mecp2^fl/y^ mice; t test, AP half-width: *t* = 0.6790, df = 19, *p* = 0.5053; rheobase: *t* = 0.1706, df = 19, *p* = 0.8663; threshold: *t* = 0.2784, df = 19, *p* = 0.7836; V_rest_: *t* = 0.4459, df = 20, *p* = 0.6605; P21-25: *n* = 12 neurons/4 PV-Cre mice; *n* = 14 neurons/4 PV-Cre::Mecp2^fl/y^ mice; t test, ap half-width: *t* = 2.279, df = 24, *p = 0.0318; rheobase: *t* = 0.3471, df = 25, *p* = 0.7314; threshold: *t* = 0.5219, df = 24, *p* = 0.6065; V_rest_: *t* = 1.264, df = 24, *p* = 0.2185). **c.** Example traces of whole-cell recordings of synaptic currents in the PNs within the LA of PV-Cre and PV-Cre::Mecp2^fl/y^ mice at P12-14 and at P21-25 (P12-14: *n* = 10 neurons/3 PV-Cre mice; *n* = 10/4 PV-Cre::Mecp2^fl/y^ mice; P21-25: *n* = 12 neurons/3 PV-Cre mice; *n* = 14/5 PV-Cre::Mecp2^fl/y^ mice). Frequencies and amplitudes of sEPSCs (freq.: two-way ANOVA, genotype: F(1, 42) = 3.672, *p* = 0.0622; ampl.: two-way ANOVA, F(1,42) = 2.573, *p* = 0.1162). The frequencies and amplitudes of sIPSCs (freq.: two-way ANOVA, F(1, 44) = 7.028, *p = 0.0111, post-hoc Sidak’s multiple comparisons test, ^#^*p* = 0.0181; ampl.: two-way ANOVA, F(1, 38) = 0.006738, *p* = 0.9350). All the data are shown as the mean ± S.E.M

In the LA, the establishment of an excitatory-inhibitory balance depends on the timely maturation of GABAergic interneurons and their synapses onto principal neurons [32]. In the developing mouse brain, PV-immunoreactive cells first appear around postnatal day 10 (P10), and adult-like PV expression patterns are established mainly by the end of the 3^rd^ postnatal week (P21) [33, 34]. To investigate the developmental trajectory of the inhibitory deficit and the elevated excitability of the LA due to *Mecp2* loss in PV interneurons, we analysed the electrophysiological properties of PNs at two time points: P12-14 and P21-25. There was no difference in the action potential firing frequencies of PNs between genotypes at any time point, with only a tendency towards elevated excitability at the later developmental point of knockout mice compared to controls (Mixed-effect model (REML), genotype effect: F (1, 17) = 0.02818, *p* = 0.8687; P21-25: REML, genotype effect: F(1, 27) = 0.9815, *p* = 0.3306) (Fig. 6a). Action potential half-width decreases during PNs postnatal maturation in the BLA [35]. There was a difference in the action potential half-width between genotypes at P21-25 (PV-Cre vs. PV-Cre::Mecp2^fl/y^: 1.595 ± 0.0705 vs. 1.377 ± 0.0646 ms (*n* = 12 and 14), t test, *t* = 2.279, df = 24, *p* = 0.0318) (Fig. 6b), with a narrower action potential detected in PNs of knockout mice as compared to controls, suggesting a faster maturation. However, there was no difference in other properties: rheobase, firing threshold, or resting membrane potential at P12-14 or at P21-25 (Supplementary Table 1) (Fig. 6b). The analysis of synaptic transmission demonstrated no difference in the developmental trajectory of sEPSCs in PNs and no differences in the currents’ frequencies or amplitudes between genotypes at any time point (freq.: PV-Cre::Mecp2^fl/y^ vs. PV-Cre at P12-14 (*n* = 10 and 10): 3.14 ± 0.594 vs. 2.53 ± 0.476 Hz; at P21-25 (*n* = 14 and 12): 3.14 ± 0.318 vs. 2.26 ± 0.182 Hz; two-way ANOVA, genotype effect: F(1, 42) = 3.672, *p* = 0.0622; ampl.: PV-Cre::Mecp2^fl/y^ vs. PV-Cre at P12-14 (*n* = 10 and 10): 10.11 ± 0.425 vs. 10.98 ± 0.683 pA; at P21-25 (*n* = 14 and 12): 10.97 ± 0.655 vs. 11.97 ± 0.568; two-way ANOVA, genotype effect: F (1, 42) = 2.573, *p* = 0.1162) (Fig. 6c). At the same time, there was a significant difference in the developmental trajectory of sIPSCs’ frequencies (Fig. 6c), but not amplitudes (freq.: PV-Cre::Mecp2^fl/y^ vs. PV-Cre at P12-14 (*n* = 10 and 10): 2.32 ± 0.407 vs. 3.14 ± 0.590 Hz; at P21-25 (*n* = 14 and 12): 1.57 ± 0.239 vs. 3.22 ± 0.586 Hz: two-way ANOVA, genotype effect: F(1, 44) = 7.028, *p* = 0.0111; ampl.: PV-Cre::Mecp2^fl/y^ vs. PV-Cre at P12-14 (*n* = 10 and 10): 10.39 ± 0.724 vs. 11.54 ± 1.119 pA; at P21-25 (*n* = 14 and 12): 11.18 ± 0.515 vs. 10.14 ± 0.481 pA, two-way ANOVA, genotype effect: F (1, 42) = 0.00674, *p* = 0.9350) (Fig. 6c). The difference in the inhibitory current frequencies was significant at the P21-25 (post-hoc Sidak’s multiple comparisons test, P21-25 between genotypes: *p* = 0.0181) (Fig. 6c). Overall, the data show that inhibitory deficit in the LA of PV-Cre::Mecp2^fl/y^ mice manifests in adolescent age around 3^rd^ postnatal week. At this age, however, the deficit is not yet sufficient to significantly affect PNs’ excitability. Additionally, at P21-25, the percentage of PV interneurons expressing *Mecp2* protein in BLA of PV-Cre::Mecp2^fl/y^ mice was 62.39 ± 6.809% (Supplementary Fig. 1c), which might not be enough to reach the same effects on the LA circuitry as in adults.

### Loss of *Mecp2* in PV interneurons affects functional connectivity in BLA-vHPC and BLA-mPFC circuits in vivo in the resting state

**Fig. 7:**
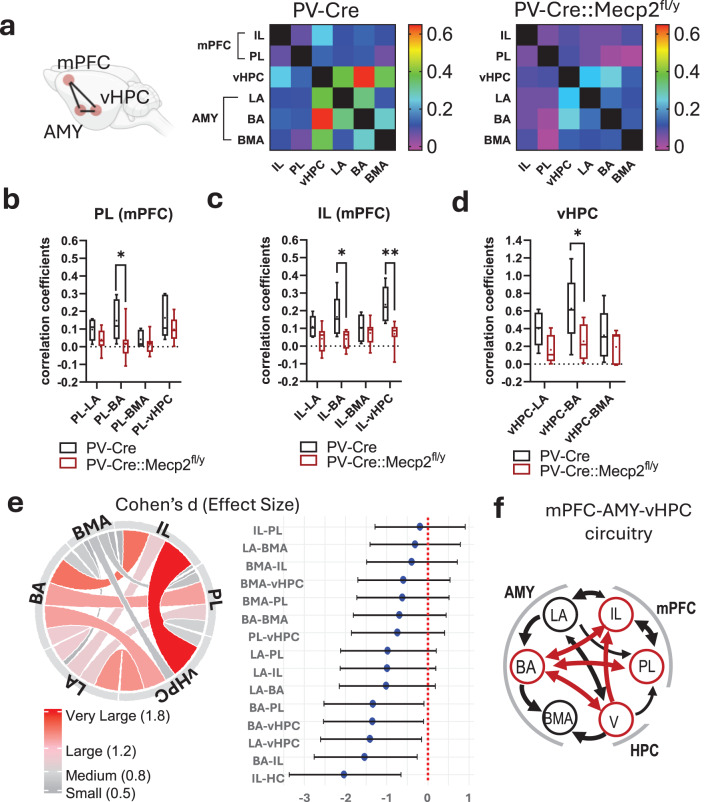
*Mecp2* gene loss in parvalbumin interneurons affects functional connectivity between the amygdala, hippocampus, and prefrontal cortex. **a.** Heatmaps of Pearson’s correlation coefficients of activity between the following brain areas of anaesthetised mice with the PV-Cre and PV-Cre::Mecp2^fl/y^ genotypes: the infralimbic (IL) and prelimbic (PL) medial prefrontal cortex, ventral hippocampus (vHPC), lateral amygdala (LA), basal amygdala (BA), and basal medial amygdala (BMA). **b.** Comparison of the Pearson's correlation coefficients between activity in the vHPC and amygdala of PV-Cre and PV-Cre::Mecp2^fl/y^ mice (*n* = 5 mice for PV-Cre, *n* = 9 mice for PV-Cre::Mecp2^fl/y^; multiple t tests, Holm‒Sidak: vHPC‒LA: *t* = 1.894, df = 36.00, *p* = 0.1282; vHPC‒BA: *t* = 2.941, df = 36.00, *p = 0.0169; vHPC‒BMA: *t* = 1.039, df = 36.00, *p* = 0.3059). **c.** Comparison of the Pearson's correlation coefficients between activity in the PL and amygdala of PV-Cre and PV-Cre::Mecp2^fl/y^ mice (*n* = 5 mice for PV-Cre, *n* = 9 mice for PV-Cre:Mecp2^fl/y^; multiple t tests, Holm‒Sidak: PL-LA: *t* = 1.338, df = 48.00, *p* = 0.3505; PL-BA: *t* = 3.089, df = 48.00, *p = 0.01327; PL-BMA: *t* = 0.7150, df = 48.00, *p* = 0.4780; PL-vHPC: *t* = 1.524, df = 48.00, *p* = 0.3505). **d.** Comparison of the Pearson’s correlation coefficients between activity in the IL and amygdala of PV-Cre and PV-Cre::Mecp2^fl/y^ mice (*n* = 5 mice for PV-Cre, *n* = 9 mice for PV-Cre::Mecp2^fl/y^; multiple t tests, Holm‒Sidak: IL‒LA: *t* = 1.619, df = 48.00, *p* = 0.2114; IL‒BA: *t* = 2.967, df = 48.00, *p = 0.01395; IL‒BMA: *t* = 0.6536, df = 48.00, *p* = 0.51651; IL‒vHPC: *t* = 3.940, df = 48.00, **p = 0.0010). **b-d.** the data are shown as 5–percentile boxes, and the mean is shown as a dot. **e.** Chord diagram of Cohen’s d sizes of effect for connectivity (colour and thickness of the links codes for effect size absolute value) and forest graph representing direction and sizes of the effects (Cohen’s d) with confidence intervals. **f.** a connectivity scheme between the BLA, mPFC and vHPC, which is based on published data [38–44]. Line width depicts the density of projections, and red arrows demonstrate connectivity that can be associated with deficits in correlated activity in PV-Cre:Mecp2^fl/y^ mice, as detected by fUS

fUS imaging has broad spatial coverage, providing large-scale measurements of brain activity in vivo [36]. The functional connectivity of the LA, BA and basomedial (BMA) amygdala nuclei with the ventral hippocampus (vHPC), prelimbic (PL) and infralimbic (IL) parts of the medial prefrontal cortex (mPFC) was analysed simultaneously via fUS imaging in anaesthetised mice. The resting-state connectivity was assayed in sagittal planes based on the calculation of the mean Pearson’s correlation factor in the fUS signal between the anatomically defined regions of interest [37]. The correlation coefficients were then Fisher-transformed and used to build the functional connectivity matrices for statistical analysis. The results demonstrated a general trend for lower amygdala resting-state connectivity with regions of interest in the brains of PV-Cre::Mecp2^fl/y^ mice (Fig. 7a). Compared with that in control littermates, the correlated activity in PV-Cre::Mecp2^fl/y^ mice was significantly lower between the BA area and vHPC (0.6272 ± 0.1720 for PV-Cre vs. 0.2570 ± 0.06707 for PV-Cre::Mecp2^fl/y^, multiple t tests, Holm‒Sidak, t ratio = 2.941, df = 36.00, *p* = 0.016960) (Fig. 7b), as was the correlated activity between the BA and both the PL and the IL parts of the mPFC (PV-Cre vs. PV-Cre::Mecp2^fl/y^: for PL‒BA, 0.1477 ± 0.05233 vs. 0.01462 ± 0.02987, multiple t tests, Holm‒Sidak, *t* = 3.089, df = 48.00, *p* = 0.01327; for the IL‒BA, 0.1650 ± 0.05335 vs. 0.04040 ± 0.01749, multiple t tests, Holm‒Sidak, *t* = 2.967, df = 48.00, *p* = 0.01395) (Fig. 7c and d). Interestingly, when the functional connectivity between the vHPC and mPFC was evaluated, it was significantly lower only in the vHPC-IL but not in the vHPC-PL pair (PV-Cre vs. PV-Cre::Mecp2^fl/y^: for the PL-vHPC, 0.1634 ± 0.05447 vs. 0.09774 ± 0.02235, multiple t tests, Holm‒Sidak, *t* = 1.524, df = 48.00, *p* = 0.3505; for the IL‒vHPC, 0.2345 ± 0.04655 vs. 0.06915 ± 0.02221, multiple t tests, Holm‒Sidak, *t* = 3.940, df = 48.00, **p = 0.0010) (Fig. 7c and d). Among statistically significant differences in connectivity, the strongest size of effect (very large, > 1.8) was for the vHPC-IL axis (Cohen’s d = −2.04, CI = 95%) (Fig. 7e). Smaller, but still large effect sizes (1.2–1.8) were evaluated for the BA connectivity with the mPFC and vHPC (Cohen’s d for BA-IL = −1.54; for BA-PL = −1.34; for BA-vHPC = −1.34, CI = 95%) (Fig. 7e).

## Discussion

The loss of *Mecp2* gene in PV interneurons recapitulates several Rett syndrome-related symptoms, including altered social interaction behaviour, fear learning, and motor dysfunction [7]. A further study demonstrated that motor dysfunction in these mice was caused by the absence of *Mecp2* in the cerebellum [45]. In the current study, we show circuit abnormalities in the vHPC-BLA-mPFC interconnected areas of PV-Cre::Mecp2^fl/y^ mice. This triad, with the BLA serving as a hub, plays a critical role in social interaction, fear learning, and fear expression [46, 47–51].

Although among PV-Cre mouse lines, the line in our study is the most widely used and has high specificity and efficiency in targeting PV interneurons in different cortical areas, a minor population of PV neurons negative for the Cre-dependent reporter gene expression has been shown before [18, 52]. This is further confirmed by the Allen Institute’s Mouse Brain Connectivity Atlas transgenic characterisation quantitative analysis [52]. Another existing PV-Cre line used in previous studies on the *Mecp2* gene loss [7] also did not target an entire brain population of PV-expressing neurons but has been reported to have comparable effectiveness and pattern of Cre-dependent reporter gene expression to the line used in our study [52]. In the brain areas evaluated in our study, *Mecp2* knockout resulted in a loss of *Mecp2* expression in the vast majority of PV interneurons, with 30% of the subset remaining MeCP2-positive. This observation in the hippocampus, amygdala, and mPFC is consistent with a previous report from the visual cortex, made in animals generated using the same PV-Cre mouse line and breeding strategy for *Mecp2* conditional knockout [18]. MeCP2 is a stable protein, and it takes time, sometimes weeks, after an onset of conditional knockout to eliminate it from neurons [53]. Taking into account that the endogenous PV promoter/enhancer drives Cre expression, a gene knockout is expected to be coincident with the start of PV expression (P10) [33, 34]. Our data are in line with previous observations [53]: the number of MeCP2+ PV interneurons of around 60% is still detectable at the 3^rd^ week after birth, which is approximately twice as high as in adult conditional knockouts. One or two weeks from the start of PV expression, by P21-25, were not enough to eliminate MeCP2 protein in the majority of PV interneurons.

Our present data demonstrate that the PV interneurons in the LA lacking the *Mecp2* gene possess synaptic abnormalities: the amplitudes of excitatory input signals are elevated, whereas the GABAergic output signal is reduced. These changes accompanied the deficit in both fast and slow inhibitory input to the LA PNs. Under these conditions, the excitability of the amygdala was elevated, whereas its functional connectivity with the vHPC and mPFC was attenuated. Overall, the results support a key role of PV interneurons in brain network malfunction related to the loss of *Mecp2*. Microcircuit malfunction associated with the general loss of *Mecp2* in neurons has been previously demonstrated in the mPFC and HPC [17, 54–56]. However, the effect on local microcircuitry has never been addressed in the BLA. *Mecp2* knockdown specifically targeted to BLA neurons has been shown to impair amygdala-dependent learning and memory [57]. Full knockout or *Mecp2* mutant expression has been shown to affect the development of cortico-LA synapses at PNs [58]. Given that the LA has a specific role in plasticity and is a key structure in fear learning, we concentrated on the local circuits involving PV interneurons in the LA.

The use of selective optogenetic stimulation revealed that GABA release from LA PV interneurons lacking *Mecp2* was significantly impaired. In addition, we observed that in PV interneurons lacking *Mecp2*, the amplitudes of spontaneous excitatory currents were greater than those in controls, while the inhibitory inputs were not altered. These findings suggest that loss of *Mecp2* upregulated glutamatergic transmission to PV interneurons, possibly via changes in the expression or cell membrane targeting of postsynaptic glutamate receptors. Association between AMPA receptor levels at synapses and *Mecp2* has been demonstrated previously in the HPC [59]. Although the excitability of PV interneurons was not altered in our ex vivo experiments, alterations in the strength of glutamatergic synapses may affect the recruitment of PV interneurons in vivo in response to the activation of afferent pathways conveying behaviourally relevant information [13, 38].

PV interneurons target the perisomatic area of PNs in the BLA and provide potent inhibition to regulate their excitability [30]. Accordingly, in line with the impaired GABA release from PV interneurons in the LA of PV-Cre::Mecp2^fl/y^ mice, we observed a deficit in spontaneous inhibitory synaptic input to PNs. The alterations in the inhibition of PNs in the LA of PV-Cre::Mecp2^fl/y^ mice involved not only fast synaptic GABA_A_ receptor currents but also slow metabotropic inhibition mediated by GABA_B_ receptors. GABA_B_ receptors activate GIRK channels in the BLA, where GIRK1, GIRK2, and GIRK3 channel subunit mRNAs are expressed [60]. There were no significant differences in the number of functional GABA_B_ receptors or recruitable GIRK channels, as the potent agonist was able to induce currents with comparable amplitudes in PNs of both genotypes. Therefore, the reduction in slow GABA_B_ receptor-mediated inhibition can also be fully attributed to the observed weaker synaptic release of GABA from PV interneurons. In line with the inhibitory deficit and elevated excitability, PNs in the LA of PV-Cre::Mecp2^fl/y^ mice fired action potentials more frequently in response to current steps.

The inhibitory synaptic deficit manifested at the 3^rd^ week of postnatal development. At this age, however, there was no difference in PNs’ excitability, with the action potential half-width being the only difference between genotypes. The action potential half-width decreases during neuron development [35]. The faster reduction in the half-width duration of PNs in the LA of PV-Cre::Mecp2^fl/y^ mice can be attributed to earlier maturation, as there was no difference in this parameter in the adult age PNs between genotypes. The mouse BLA PNs’ membrane properties and excitability are still developing at the 3^rd^ week after birth [35]. This, and only around 40% reduction in PV interneurons expressing MeCP2 protein at this age, might contribute to the absence of the major effect on PNs excitability, visible in adults.

Synchronous oscillatory activity between the vHPC, BLA, and mPFC is present during social interaction, fear and memory performances [46, 61, 51, 62], as reviewed previously in [63, 50]. Numerous evidence show that PV interneurons play a unique role within brain networks, efficiently synchronising the activity of postsynaptic PNs through their strong inhibitory synapses and extensive axonal arborisation [64–66]. Accordingly, PV interneurons provide feedforward inhibition, regulate behaviourally meaningful network oscillatory activities in the BLA, and coordinate network synchronisation across brain regions [64–66]. In vivo recordings suggest that projections from the mPFC and vHPC directly innervate local interneurons in the BLA to recruit feedforward inhibition and control action potential generation in PNs [38, 46]. The observed synaptic abnormalities within the PV interneurons of the amygdala can, therefore, interfere with these processes by receiving incorrect input signals and providing weaker inhibition.

In vivo fUS imaging revealed a significant reduction in the resting-state functional connectivity of the BA with the mPFC and vHPC in PV-Cre::Mecp2^fl/y^ mice compared with their PV-Cre littermates. Moreover, we observed a reduction in correlated activity between the mPFC (IL) and vHPC, demonstrating that all three nodes of the mPFC-BLA-vHPC triad were affected. Previously, it has been demonstrated that full *Mecp2* knockout or duplication induces abnormalities in oscillatory activity and synchronisation in the mPFC and HPC [55, 58, 67, 68]. Additionally, malfunction of vHPC projections to the mPFC has been shown in *Mecp2* full-knockout mice [69]. These previously reported data align with our findings on connectivity, suggesting that *Mecp2* loss in PV interneurons may play a central role in the observed effects. However, as neurons of PV-Cre::Mecp2^fl/y^ mice exhibit knockout in all three brain areas, it is impossible to claim that one local population of PV neurons in a specific brain area contributes more than in another brain area when resting-state correlated neuronal activity is evaluated.

The pattern of connectivity deficits we observed was associated with specific subdivisions within the analysed brain areas. This can be explained by the connectivity pattern within the mPFC-BLA-vHPC triad, which was described by tracing and optogenetic analyses in previously published studies (Fig. 7f). Amygdala PNs projecting to the vHPC are evenly distributed across the LA and BA, while the vHPC innervates the BA and BMA more densely than the LA [39]. The LA projects to the BA, where its synchronous activity with vHPC afferents is crucial for learning-related plasticity at LA-BA synapses of PNs [38]. The connections between the mPFC and vHPC are not reciprocal. vHPC inputs innervate both the IL and PL regions of the mPFC, although PL neurons receive significantly smaller inputs than those in the IL [40]. The BLA forms reciprocal connections with both the PL and the IL regions of the mPFC. Projections from the IL are biased toward the LA, whereas those from the PL are biased toward the BA [41–43, 70]. Notably, the PL is reciprocally connected with the BA, targeting PNs that, in turn, project to the vHPC [44]. Meanwhile, this convergence of inputs at the same PNs is absent in the LA and BMA [44]. Thus, the observed functional connectivity deficits were detected between the most anatomically and functionally interconnected areas of the mPFC-BLA-vHPC triad (Fig. 7f), where convergence of afferents and reciprocal nature of connections likely amplify the impact of *Mecp2* loss in PV neurons, leading to widespread disruption of network correlated activity.

In rodents, a network linking the mPFC-BLA-vHPC underlies multiple aspects of aversive and social behavior, memory consolidation and extinction [58–72]. In particular, the network is involved in the contextual regulation of fear expression [71]. During cued fear expression, theta oscillations synchronize BLA with the HPC and mPFC [61]. Moreover, the coordinated mPFC-BLA oscillatory activity changes during social behavior. This pattern of activity can be replicated by stimulating PV interneurons in mPFC, which can restore social behavior in the Shank3-related mouse model of ASD [72]. Therefore, the previously reported behavioral abnormalities in mice with conditional *Mecp2* knockout in PV interneurons [7]: changes in social interaction and cued memory – are consistent with the observed pattern of reduced functional connectivity within the mPFC-BLA-vHPC triad.

Malfunction of PV interneurons has been previously shown to be associated with ASD and other neurodevelopmental conditions [9, 10, 73]. Further studies on the mechanistic link between the functional impairment of these neurons, effects on circuits, brain connectivity and phenotypes are warranted to understand the alterations observed in those animal models, including *Mecp2*-related pathologies.

### Limitations

First, although evidence of endophenotype replication and drug treatment responses supported the validity of the mouse model for studying Rett syndrome-related pathology [4, 74–76], the results obtained from mice may not always translate to humans. Second, this study used only male mice, even though Rett syndrome affects mainly girls. However, the use of male mice is usual and scientifically justified for mechanistic studies of *Mecp2* function. The use of male mutants enables controlled mechanistic interrogation of *Mecp2* loss without the additional variability introduced by X-inactivation mosaics. Male mouse models of Rett syndrome show robust, reproducible circuit and synaptic phenotypes that closely mirror those detected in female heterozygous mutants or knockouts [4, 75, 77]. Therefore, using male conditional knockouts provides a stable and interpretable system for dissecting developmental and circuit mechanisms, especially when targeting a single cell type, while still yielding results relevant to and translatable to the female Rett syndrome phenotype. Moreover, since previous studies in the field used knockout male mice [7, 18], we aimed to advance the existing body of research to understand the consequences of *Mecp2* gene loss in parvalbumin-expressing interneurons in the mouse brain with the same approach. In our study, the conditional deletion affects only a subset of parvalbumin interneurons, which further approximates the mosaic cellular pattern in females while maintaining experimental stability.

## Conclusions

We demonstrated that PV neuron-specific loss of the *Mecp2* gene led to amygdaloid microcircuit abnormalities, that originated in late postnatal development. This was mechanistically evidenced by an increased amplitude of excitatory drive and a reduction in GABA release in PV interneurons in the amygdala. The inhibitory signals provided by PV interneurons are known to be necessary for the efficient orchestration of behaviourally relevant neuronal network activities, which provides system-level significance for these results. At the larger network level, we found that the lack of the *Mecp2* gene in PV interneurons leads to weakened correlated activities between the amygdala and reciprocally connected brain areas in vivo. A weakening of resting-state functional connectivity was observed within the entire mPFC-BLA-vHPC network triad, indicating the critical role of *Mecp2* in the ability of PV interneurons to coordinate network activity between these structures.

## Electronic supplementary material

Below is the link to the electronic supplementary material.

Supplementary Material 1

## Data Availability

All data and data analysis associated with this study are available upon request.

## Abbreviations

AP: Action potential
AAV: Adeno-associated virus
BA: Basal amygdala
BLA: Basolateral amygdala
BMA: Basomedial amygdala
ChR2: Channelrhodopsin-2
IL: Infralimbic cortex
fUS: Functional ultrasound
GIRK: G protein-coupled inwardly rectifying potassium channel
LA: Lateral amygdala
MECP2: Methyl CpG binding protein 2
mPFC: Medial prefrontal cortex
PL: Prelimbic cortex
PN: Principal neuron
PV: Parvalbumin
sEPSC: Spontaneous excitatory post-synaptic current
sIPSC: Spontaneous inhibitory post-synaptic currents
vHPC: Ventral hippocampus
