# Supplementary material for: Loss of the Mecp2 gene in parvalbumin interneurons leads to an inhibitory deficit in the amygdala and affects its functional connectivity
Source: Mol Autism. 2026 Jan 5;17:4. doi: 10.1186/s13229-025-00699-5 (PMC12829049; doi:10.1186/s13229-025-00699-5)
Supplement: Supplementary file 1 — Supplementary Material 1 [file 13229_2025_699_MOESM1_ESM.docx]

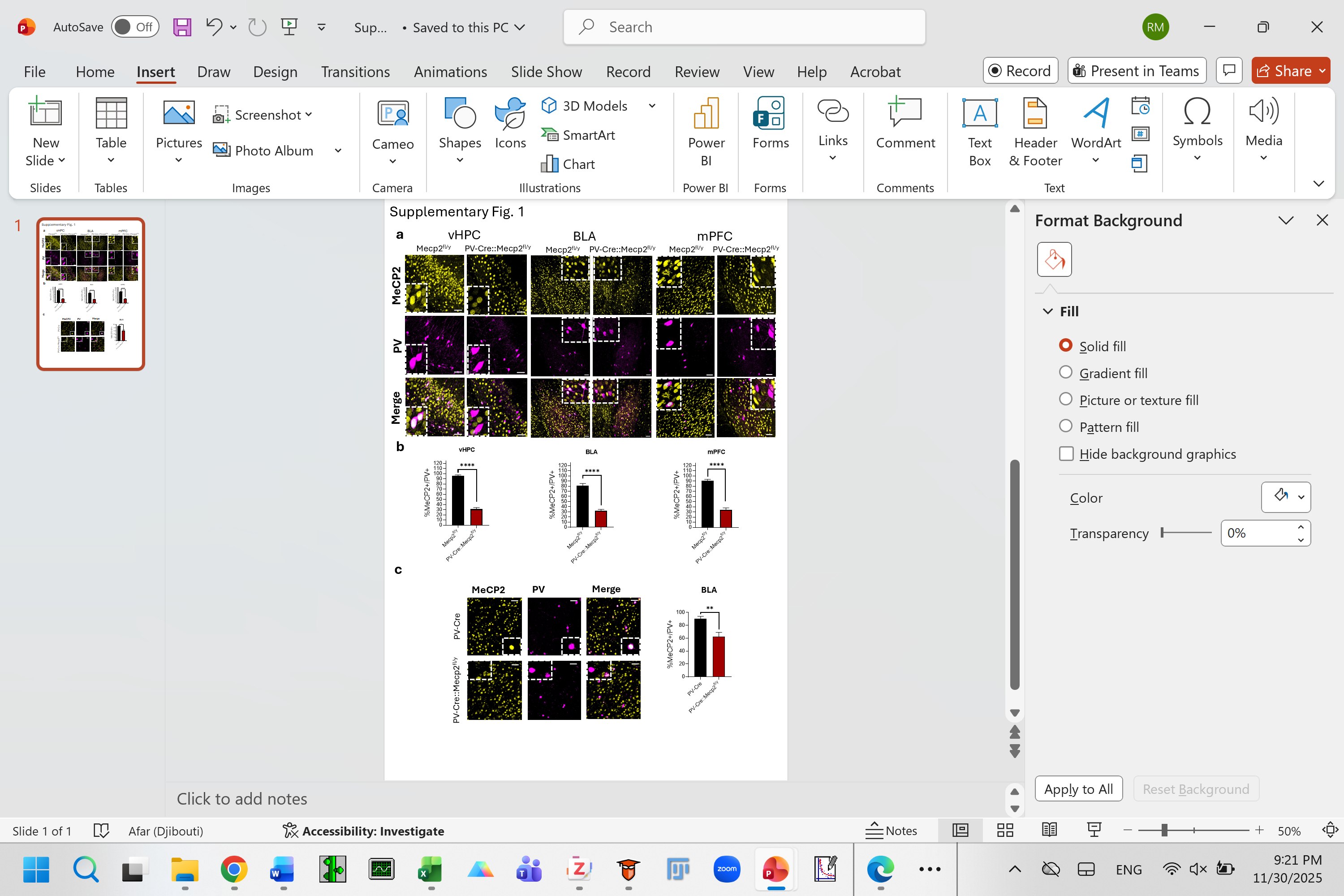


**Supplementary Fig. 1. Conditional knock-out of the Mecp2 gene in parvalbumin interneurons of adult and adolescent male mice. a.** Microscopy images of IHC staining demonstrating PV neurons lacking MeCP2 protein in vHPC, BLA and mPFC brain slices of PV-Cre::Mecp2^fl/y^ mice after conditional knockout, scale bar is 50 µm, inserted images are 3x magnification. **b.** Percent of parvalbumin interneurons (PV+) positive for MeCP2 protein (MeCP+) in brain slices of PV-Cre::Mecp2^fl/y^ and Mecp2^fl/y^ adult mice based on IHC images analysis. 31.08 ± 3.125% of PV interneurons are still positive in vHPC (Mecp2^fl/y^ vs. PV-Cre::Mecp2^fl/y^: 96.02 ± 1.635% vs. 31.08 ± 3.125%, n=15 brain slices / 4 Mecp2^fl/y^ mice and n=14 brain slices / 3 PV-Cre::Mecp2^fl/y^ mice; Mann-Whitney test, U=0, ****p<0.0001), 32.40 ± 2.300% – in BLA (Mecp2^fl/y^ vs. PV-Cre::Mecp2^fl/y^: 80.57 ± 4.628% vs. 32.40 ± 2.300%, n=19 brain slices / 4 Mecp2^fl/y^ mice and n=15 brain slices / 3 PV-Cre::Mecp2^fl/y^ mice; Mann-Whitney test, U=12, ****p<0.0001), and 34.03 ± 3.870% – in mPFC (Mecp2^fl/y^ vs. PV-Cre::Mecp2^fl/y^: 90.96 ± 2.334% vs. 34.03 ± 3.870%, n=18 brain slices / 4 Mecp2^fl/y^ mice and n=10 brain slices / 3 PV-Cre::Mecp2^fl/y^ mice; Mann-Whitney test, U=0, ****p<0.0001). **c.** Microscopy images of IHC staining demonstrating PV neurons lacking MeCP2 protein in BLA brain slices of P21-25 PV-Cre::Mecp2^fl/y^ mice after conditional knockout, scale bar is 50 µm, inserted images are 3x magnification. Percent of parvalbumin interneurons (PV+) positive for MeCP2 protein (MeCP+) in brain slices of PV-Cre and PV-Cre::Mecp2^fl/y^ P21-25 mice based on IHC images analysis: 90.12 ± 3.888 vs. 62.39 ± 6.809 (n=16 brain slices from 3 PV-Cre::Mecp2^fl/y^ mice and n=17 brain slices from 3 PV-Cre mice) (Mann-Whitney test, U=52.5, **p=0.0013)

**Supplementary Table 1.** **Action potential firing properties in neonatal and adolescence PV-Cre and PV-Cre::Mecp2^fl/y^ mice.**

| Properties | Age/genotype | PV-Cre | PV-Cre::Mecp2^fl/y^ | t test |
| --- | --- | --- | --- | --- |
| AP half-width | P12-14 | 1.827 ± 0.1873 ms (n=8) | 1.996 ± 0.1576 ms (n=13) | t=0.6790, df=19, p=0.5053 |
|  | P21-25 | 1.595 ± 0.0705 ms (n=12) | 1.377 ± 0.0646 ms (n=14) | t=2.279, df=24, *p=0.0319 |
| Rheobase | P12-14 | 42.5 ± 4.605 pA (n=8) | 43.85 ± 6.748 pA (n=13) | t=0.1706, df=19, p=0.8663 |
|  | P21-25 | 77.69 ± 7.817 pA (n=13) | 73.57 ± 8.998 pA (n=14) | t=0.3471, df=25, p=0.7314 |
| Threshold | P12-14 | -37.92 ± 1.601 mV (n=8) | -37.35 ± 1.259 mV (n=13) | t=0.2784, df=19, p= 0.7836 |
|  | P21-25 | -36.29 ± 3.203 mV (n=12) | -34.34 ± 2.130 mV (n=14) | t=0.5219, df=24, p=0.6065 |
| V_rest_ | P12-14 | -53.55 ± 3.027 mV (n=8) | -55.13 ± 2.048 mV (n=14) | t=0.4459, df=20, p=0.6605 |
|  | P21-25 | -62.57 ± 1.401 mV (n=11) | -59.99 ± 1.412 mV (n=15) | t=1.264, df=24, p=0.2185 |
